# Improving Individualized Salbutamol Treatment: A Population Pharmacokinetic Model for Oral Salbutamol in Virtual Patients

**DOI:** 10.3390/pharmaceutics17010039

**Published:** 2024-12-30

**Authors:** Lara Marques, Nuno Vale

**Affiliations:** 1 PerMed Research Group, Center for Health Technology and Services Research (CINTESIS), Rua Doutor Plácido da Costa, 4200-450 Porto, Portugal; 2 CINTESIS@RISE, Faculty of Medicine, University of Porto, Al. Prof. Hernâni Monteiro, 4200-319 Porto, Portugal; 3 Department of Community Medicine, Health Information and Decision (MEDCIDS), Faculty of Medicine, University of Porto, Rua Doutor Plácido da Costa, 4200-450 Porto, Portugal

**Keywords:** salbutamol, popPK modeling, PBPK modeling, pharmacokinetics, covariates, interindividual variability, personalized medicine

## Abstract

Background: Salbutamol, a short-acting β_2_-agonist used in asthma treatment, is available in multiple formulations, including inhalers, nebulizers, oral tablets, and intravenous, intramuscular, and subcutaneous routes. Each formulation exhibits distinct pharmacokinetic (PK) and pharmacodynamic (PD) profiles, influencing therapeutic outcomes and adverse effects. Although asthma management predominantly relies on inhaled salbutamol, understanding how these formulations interact with patient-specific characteristics could improve personalized medicine approaches, potentially uncovering the therapeutic benefits of alternative formulations for an individual patient. Herein, this study aims to analyze how covariates—such as age, weight, gender, body surface area (BSA), cytochrome P450 (CYP) expression, race, and health status—affect the therapeutic regime of orally administered salbutamol using population PK (popPK) modeling. The final model is intended as a tool to support the selection of optimal formulation and dosage regimen based on individual patient profiles. Methods: A dataset of 40 virtual patients derived from a physiologically based PK (PBPK) model of oral salbutamol was included in the popPK model. Results: A two-compartment model with first-order elimination and absorption, with a transit compartment, best described the plasma concentration–time profile following a 4 mg dose. Relationships were identified between gender and mean transit time (M_tt_) and clearance (C*l*), as well as the effects of weight and BSA on the volume of distribution of the central compartment (V1) and C*l*, and a significant impact of health status on C*l*. Conclusions: Despite current contraindications for oral salbutamol, our findings suggest that certain individuals, particularly children, may benefit from oral treatment over inhalation. We also identified individual characteristics associated with increased salbutamol toxicity risk, indicating the need for dose adjustment or alternative administration. This study further highlights the potential of popPK modeling in personalized therapy through a fully in silico approach.

## 1. Introduction

Asthma is a heterogeneous chronic disease of the lower airways, characterized by airflow limitation, airway hyperresponsiveness, and structural changes within the respiratory tract [1,2,3]. Recent data from 2019 estimate a global prevalence of 262.41 million cases, reflecting an upward trend worldwide [1]. Asthma management principles are well-established in clinical guidelines, with first-line treatment consisting of inhaled short-acting β_2_-agonists (SABAs), including salbutamol [4]. Salbutamol, a selective β_2_-adrenergic receptor agonist, is indicated for the symptomatic relief of bronchospasm by binding to β_2_ receptors and inhibiting bronchial smooth muscle contraction [5,6,7,8,9]. The pharmacokinetic (PK) and pharmacodynamic (PD) profiles of salbutamol have been extensively documented over decades of clinical use [10], emphasizing its versatility across multiple routes and formulations.

Although primarily administered via inhalation for its rapid and localized effect, salbutamol can also be given intravenously (IV), intramuscularly (IM), subcutaneously, and orally [11]. Within each route, further variations in formulations exist; for instance, oral salbutamol is available as both a syrup and a tablet. Understanding how these different formulations interact with patient-specific characteristics can provide insight into optimizing treatment strategies, particularly as salbutamol’s PK (absorption, distribution, and elimination) and PD (drug response) characteristics can vary significantly depending on it. Significant knowledge gaps remain regarding the orally administered salbutamol, particularly in understanding its systemic PK features, variability across patient populations, and lack of optimized dosing strategies. Even when a drug has optimal physicochemical properties under normal physiological conditions, its PK features are directly affected by the route of administration [12,13]. Thus, a personalized approach becomes particularly essential when multiple administration options exist, as with salbutamol, since there will indeed be individual characteristics that benefit from the use of one formulation over another. Tailoring salbutamol therapy to fit a patient’s unique profile could ultimately improve therapeutic outcomes.

In our previous work, we examined interindividual variability (IIV) in salbutamol administered as a dry powder inhaler (DPI) and found that patient-specific characteristics significantly impact the drug’s course through the body [14]. Building on this foundation, this current study aims to assess the influence of several covariates (age, weight, cytochrome P450 (CYP) expression, health status, and race) on the PK parameters of oral salbutamol. This model is intended to support the selection of optimal formulations and dosing regimens based on individual patient profiles. To do so, this research adopts a fully in silico approach, from data generation to analysis. A physiologically based PK (PBPK) model for oral salbutamol is developed to simulate treatment, following therapeutic regimens recommended by clinical practice guidelines, in a diverse cohort of virtual patients. These synthetic data are then analyzed through population PK (popPK) modeling to quantify the effects of covariates on key PK metrics. PopPK studies are valuable tools for identifying factors that influence optimal treatment regimens, and their insights contribute to advancing personalized medicine. Integrating PBPK modeling with popPK modeling offers a novel framework to better understand the PK variability and support the selection of optimal formulations based on individual patient profiles. Individualized approaches to drug therapy, which consider not only disease pathophysiology but also physiological, anatomical, and demographic factors, represent a meaningful step toward therapeutic success.

## 2. Materials and Methods

### 2.1. PBPK Model Development

**Figure 1:**
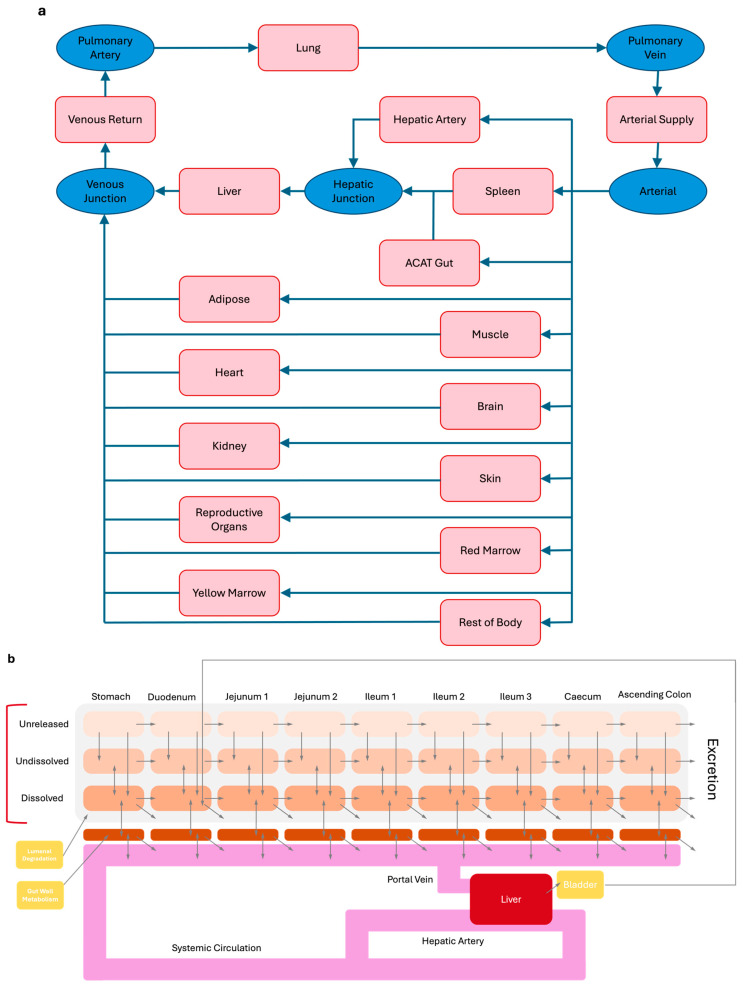
PBPK model developed for oral salbutamol, consisting of (**a**) systemic model, representing 13 tissues, and (**b**) advanced compartmental absorption and transit (ACAT) model, representing the gastrointestinal tract.

A whole-body PBPK model (Figure 1) was implemented in the GastroPlus software (Version 9.9; Simulation Plus Inc., Lancaster, CA, USA) and was used to generate virtual patient PK data. This model was constructed using values extracted from the literature, or when some parameters were unavailable in published studies, they were estimated and optimized using the ADMET Predictor^®^ (Version 12.0; Simulation Plus Inc., Lancaster, CA, USA). The drug-specific input parameters for salbutamol are depicted in Table 1. Other properties used for the PBPK predictions are provided in the Supplementary Material (Table S1).

The PBPK model for salbutamol consisted of 13 tissue compartments, including lungs, adipose, muscle, liver, spleen, heart, brain, kidneys, skin, reproductive organs, red marrow, yellow marrow, and the rest of the body. Each compartment was defined by its volume, an associated tissue blood flow rate, and tissue-to-plasma partition coefficients (K_p_) derived from ADMET Predictor^®^ and Boger et al. [15] (Table 2). Perfusion rate-limited distribution was assumed to apply to all tissues.

The absorption process of salbutamol was simulated using the advanced compartmental absorption and transit (ACAT) model, with human–physiological–fasted conditions used as the GastroPlus default. Salbutamol can be administered in fasted or fed conditions via the oral route. The oral administration (immediate-release tablet) of 4 mg of salbutamol was initially simulated as a single dose in a healthy 30-year-old American male. PK parameters were estimated using ADMET Predictor^®^, and values from previously published studies were optimized (Table 3).

### 2.2. Virtual Population Generation

As PBPK predictions aligned with the reference values, the PK profile of 4 mg of orally administered salbutamol every 6 h (4 mg q6h) was modelled in a virtual population with specific individual characteristics, including age, weight, race, gender, and health status. This population was created through the GastroPlus’ Population Simulator interface, with the generation of random population estimates for age-related (PEAR) physiologies. Specifically, four groups with different ethnicities were established: Americans, Asians, and Chinese, aged between 5 and 65 years. The body mass index (BMI) scale (BMI of 18.5–24.9 is normal, BMI of 25–29.9 is overweight, and BMI ≥ 30 is obese) was employed to determine weight and body surface area (BSA). Three groups consisted of healthy individuals without any concurrent pathological conditions, while the fourth group included patients with cirrhosis A. The male–female proportion was about 50%, except for the group with the associated comorbidity, which was 60%, as this condition predominantly affects males. The detailed characteristics of the virtual population are summarized in Supplemental Table S2.

The drug disposition-based parameters were simulated over 24 h. Demographic and PK data, particularly the plasma concentration–time profile of salbutamol, were extracted to generate a dataset for popPK modeling. The accuracy of the predicted PK parameters was evaluated based on the fold-error, and predictions were considered reliable if the fold-error was <2. This fold-error was calculated as follows: when the observed value was less than the predicted value, fold-error = predicted/observed; when the observed value was greater than the predicted value, fold-error = observed/predicted [16].

Data were initially analyzed using non-compartmental analysis (NCA), using PKAnalix (Version 2024R1, Lixoft, Antony, France). This software accurately estimates key PK parameters, providing a robust preliminary understanding of the drug’s disposition in the body. The integral method employed was linear trapezoidal linear, with equal weighting assigned to each data point. The selection of data points for the terminal phase was guided by adjusted R^2^ as the acceptance criterion, allowing for calculating the slope of the linear regression to determine λ_z_ (elimination rate constant).

### 2.3. Population Pharmacokinetic Modeling

The extraction of PK data generated through PBPK modeling resulted in a dataset of 40 PK profiles and a total of 838 observations. The population PK analysis was performed using a nonlinear mixed-effects (NLME) approach in Monolix (Version 2024R1, Lixoft, Antony, France). The popPK parameters were estimated by computing the maximum likelihood using the stochastic approximation expectation maximization (SAEM) algorithm. One-, two-, and three-compartment models, following first- or zero-order absorption, with or without lag time for extravascular-administered salbutamol (oral tablet), were initially fitted to the concentration–time data without any covariates. The PK parameters were assumed to follow a normal distribution with a combined (additive + proportional) error model. Initial estimates were automatically computed by the software, using all individuals included in this study, selecting the initial population parameters that best fit the data points.

The most appropriate structural model was systematically selected based on the lowest corrected Bayesian information criterion (BICc), alongside visual inspection of the goodness-of-fit (GOF) plots and acceptable relative standard errors (RSE) of <30% for fixed-effect estimates and <50% for random-effect estimates, ensuring accurate population mean estimates.

### 2.4. Covariate Model

Afterwards, eight potential covariates were selected based on their theoretical relevance and biological plausibility, and their effects on the PK parameters were evaluated. These covariates included the continuous variables—age, weight, BSA, and CYP2D6 and CYP2C19 expression—as well as the categorical data—gender, race, and health status.

The covariate screening process was conducted through two different approaches. The first method involved a statistical assessment using Pearson and Spearman’s correlations for continuous covariates and ANOVA tests for categorical covariates. A significance threshold of 0.05 was applied to determine whether a statistically significant correlation existed between the variable and the PK parameter under investigation. Then, a statistical test for collinearity was applied to the covariates identified as potentially impacting the PK parameters. Covariates with a variance inflation factor (VIF) > 15, indicating multicollinearity, were reconsidered in the covariate submodel analysis. In cases of multicollinearity, the covariate with the less significant *p*-value was excluded. Following this, a combined stepwise forward selection and backward elimination approach was used. The order of covariate inclusion into the base model was based on their clinical relevance and the smallest *p*-value from the previous tests. Covariate inclusion in the full model during forward selection was based on a reduction in the –2 log likelihood (LL) > 3.85 (*p* < 0.05). Covariates were added until no further improvement in the model was observed, as indicated by an increase in –2LL. Covariate selection was finalized by backward elimination, where covariates were removed one by one from the full model, starting with the one with the highest *p*-value. They were removed if the increase in –2LL was <10.83 (*p* < 0.001).

The second approach involved an automatic covariate model-building algorithm implemented in Monolix (Version 2024R1), specifically the conditional sampling use for stepwise approach based on correlation tests (COSSAC). Briefly, this method uses samples from the posterior conditional distribution to calculate the correlation between random effects and covariates. *p*-values, derived from Pearson’s correlation tests for continuous data and ANOVA tests for categorical data, are used to sort all random effect–covariate relationships, regardless of their incorporation into the model. The forward and backward stepwise methods were then employed to select covariates, as described previously. The acceptance or rejection of a covariate relationship was again based on the –2LL and BICc criteria. If these criteria were not improved, the model was not retained [17].

### 2.5. Predictive Performance Assessment of the Model

A visual predictive check (VPC) using a 90% prediction interval was performed to graphically assess potential misspecifications in structural, variability, and covariate models. Additionally, a visual inspection of the GOF plots was conducted to evaluate the model’s predictive performance, including observed vs. individual predictions, individual weighted residuals (IWRES) vs. time/predictions, and the distribution of the empirical and theoretical standard Gaussian probability density functions (PDF) against IWRES and normalized prediction distribution errors (NPDEs). The BICc was the primary criterion for evaluating model fit. Acceptable RSEs were also examined.

## 3. Results

### 3.1. PBPK Model Development and Validation

**Figure 2:**
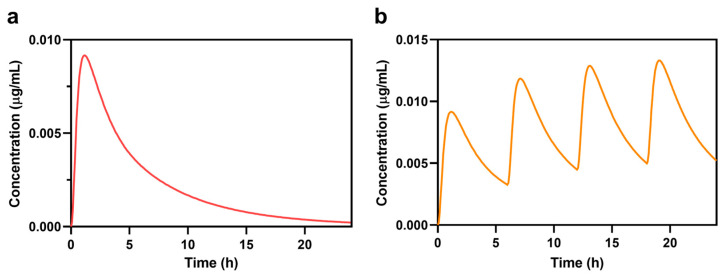
Simulation of single-dose treatment with 4 mg of oral salbutamol (**a**) and treatment with 4 mg of orally administered salbutamol every 6 h (**b**) in a healthy 30-year-old American male individual.

The observed and PBPK model-simulated plasma concentration–time profile of salbutamol was derived from a healthy 30-year-old American male (standard GastroPlus model) following a single 4 mg dose (Figure 2). The predicted PK parameters were consistent (within a <2-fold error) with reference values (observed values), as shown in Table 4. Additionally, PK parameters such as C*l*, V_d_, and t_1/2_ were also coherent. The reference values were 43.80 for C*l*, 195.75 for V_d_, and 2.78 for t_1/2_, while the model used 34.42, 167.02, and 3.36 values, respectively.

With the predicted plasma concentration–time profile matching the observed profile, the dosing regimen for simulation in the virtual population was adjusted to 4 mg every 4 h, aligning with the clinically recommended dose for asthma patients.

### 3.2. Virtual Population

A total of 838 plasma concentration observations from 40 patients (16 females, 24 males) were extracted through PBPK modeling and included for popPK model building. Patient characteristics are summarized in Table 5.

### 3.3. PopPK Model

#### 3.3.1. Base Model Selection and Full Multi-Covariate Model

The model that best fits the oral salbutamol observations was a two-compartment model with first-order absorption including transit compartments and linear elimination (BICc = –11,936.81 and –2LL = –12,022.77). The model was parameterized in terms of k_a_, transit compartments (mean transit time, M_tt_, and transit rate, K_tr_) to describe the delay in drug absorption onset, apparent clearance (C*l*/F), apparent intercompartmental clearance (Q/F), and apparent volumes of distribution for the central (V1) and peripheral (V2) compartments. A normal distribution was used to describe IIV. A combined error model with constant and proportional terms was applied to describe residual error according to the following equation:

$$\Upsilon =\left(F+\sqrt[{2}]{a^{2}}+b^{2}\cdot F\right)\cdot \varepsilon$$

where Υ is the observed concentration, *F* is the model-predicted concentration using individual parameters, *a* and *b* are constants, and *ε* is a standardized Gaussian random variable.

#### 3.3.2. Final Model

The covariate analysis involved two different methods, as described in Section 2.4. The manual approach yielded the best results (Supplementary Material) based on criteria for selecting the best model and the plausibility of included correlations. Incorporating covariates significantly improved the structural model. The manual covariate screening method identified potential gender effects on M_tt_ and C*l*, a significant impact of weight on V1 and C*l*, BSA effects on V1 and C*l*, and health status on C*l*. Parameter estimates for the final model are presented in Table 6. All estimated population parameters demonstrated RSE < 30% for fixed effects, except for C*l* and V1, and RSE < 50% for IIV. No significant correlations were found between PK parameters.

**Figure 3:**
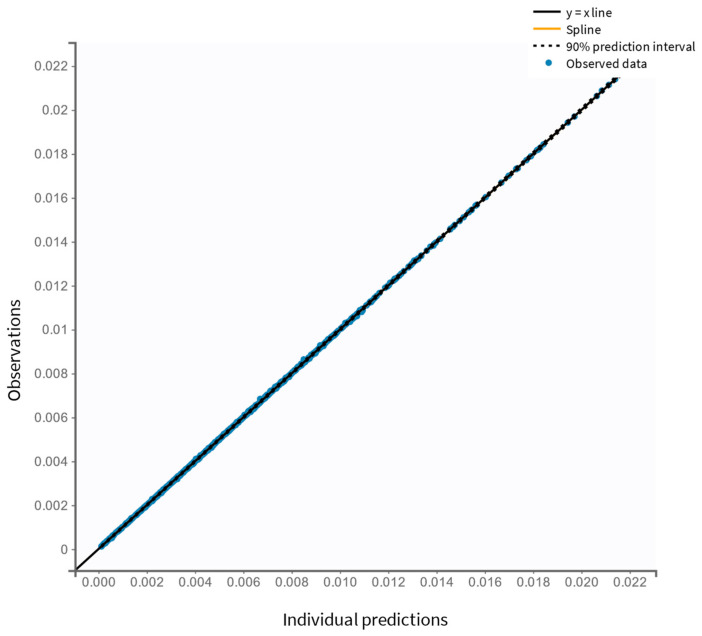
Observed salbutamol concentration (μg/mL) versus individual predictions (μg/mL) to diagnose the final popPK model.

**Figure 4:**
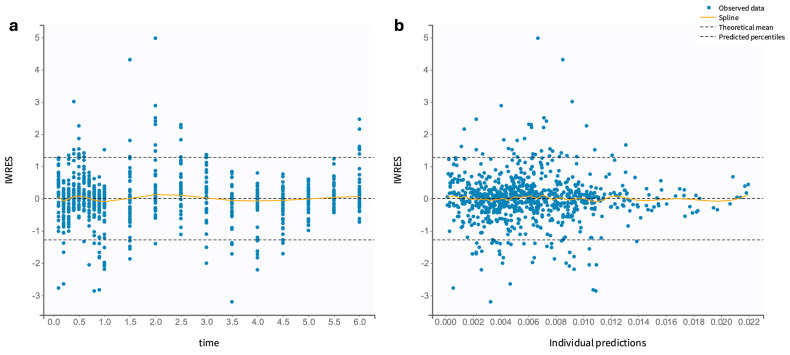
IWRES for the final popPK model: (**a**) IWRES versus time and (**b**) IWRES versus individual predicted concentrations (μg/mL). The theoretical mean corresponds to the horizontal line y = 0.

**Figure 5:**
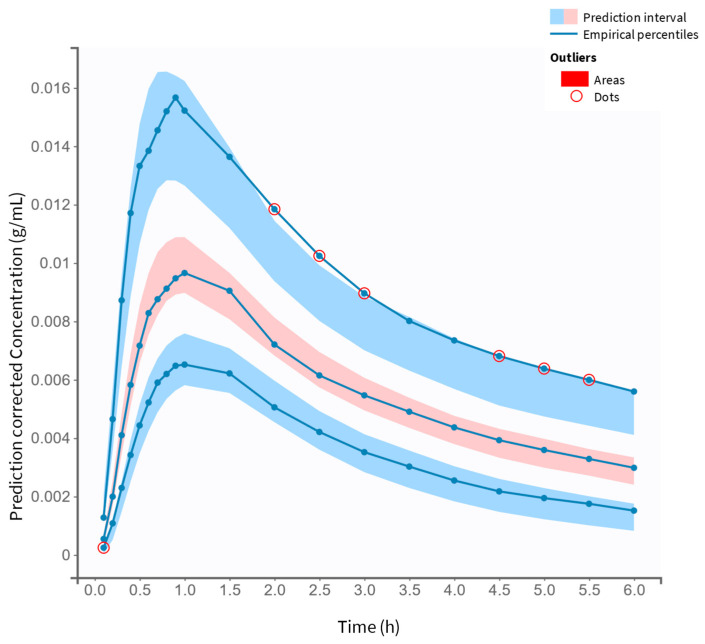
VPC for the two-compartment model with first-order absorption including transit compartments and linear elimination. The VPC of the final covariate PK model displays the prediction intervals for the 10th, 50th, and 90th percentiles (shaded areas, from bottom to top, respectively). Outliers are visualized as red dots and areas.

The final model was assessed using statistical testing and diagnostic plots. Pearson’s and Wald’s tests identified significant covariate effects (*p* < 0.05), highlighting their relevance. According to GOF plots, the model provided accurate estimates. A close alignment between the predicted and observed plasma salbutamol concentrations is detected (Figure 3), with individual predictions (calculated using empirical Bayes estimates, EBEs) centered symmetrically around the identity line (y = x). The outlier proportion was minimal at 4.89%. The low deviation from the 90% prediction interval suggests minimal bias and a high level of predictive performance for the model. The distribution of IWRES vs. time and vs. predicted concentrations (Figure 4) shows no apparent trends, as data points are evenly scattered around the zero baseline. The final model’s VPC plot (Figure 5) further confirms a strong alignment between observed and predicted salbutamol concentrations, with a minimal proportion of outliers. This overall diagnostic assessment provides insight into potential model misspecifications, though aside from minor variations in C*l* and V1 estimates, the final model demonstrates an adequate fit for describing the PK profile of orally administered salbutamol.

### 3.4. Influence of Patient-Specific Covariates on Oral Salbutamol

**Figure 6:**
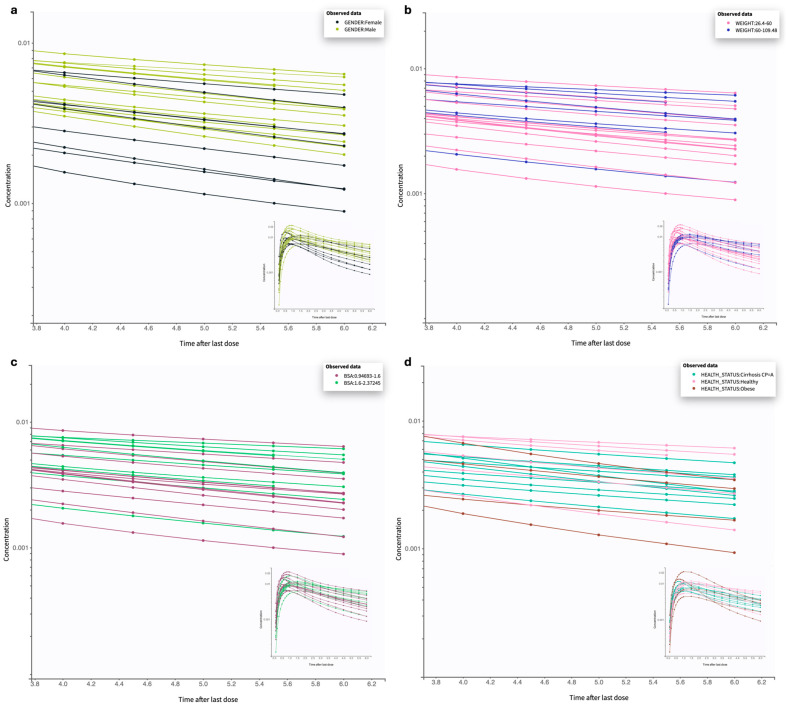
Analysis of covariates’ effect on C*l*. Evaluation of the terminal phase of the concentration-time profile of the virtual population of healthy adults (20–69 years) under oral treatment with 4 mg of salbutamol. Panel (**a**) shows the analysis stratified by gender, (**b**) by body weight, (**c**) by BSA, and (**d**) by health status, with only American individuals selected.

To explore the impact of individual covariates on the PK of oral salbutamol, previously identified correlations from popPK modeling were examined. Table 7 presents the numerical values for the M_tt_, V1, and C*l* across different subgroups defined by gender, BSA, and weight. The geometric mean and respective SD were calculated from compartmental analysis. Furthermore, the descending phase of the concentration vs. time curve (Figure 6), corresponding to the drug’s elimination phase, allows for a visual comparison of C*l* among various population subgroups, including gender, BSA, weight, and health status. Only covariates identified as potential influencers of PK parameters were included in this analysis. Additionally, the analysis was restricted to healthy adults, except in the health status subgroup, where the sample was further limited to American participants, to minimize confounding effects on this parameter.

## 4. Discussion

In this study, a popPK model for oral salbutamol was developed using a virtual population. The methodology for generating a virtual patient database followed the approach of our previous research, which demonstrates the reliability of virtual data in exploring IIV in PK parameters [14]. Therefore, a virtual population was created due to the limited availability of orally administered salbutamol data.

A two-compartment disposition model with first-order elimination and absorption, with a transit compartment, was fitted to the plasma concentrations from 40 subjects. The two-compartment model was considered appropriate, as the plasma concentration–time course exhibited a biphasic decline (two distinct slopes), as shown in Figure 5. Since salbutamol is a molecule with a relatively broad distribution, a model with a central compartment (plasma and highly perfused tissues such as kidneys, heart, and lungs) and a peripheral compartment (tissues with slower distribution, such as adipose tissue, skin, and muscle) is likely logical. Acute oral administration of salbutamol is reported to enhance muscle strength [18], demonstrating the compound’s effect on low-perfusion tissues. First-order absorption and elimination kinetics are expected for oral drugs, suggesting a direct proportionality between the absorption/elimination rate and drug concentration. Finally, the model accounts for the delay between salbutamol administration and its detection in systemic circulation as a multi-step process [19]. The transit compartment approach better described the underlying physiology, as well as the impact of the drug formulation and its physicochemical properties on the absorption process, especially since introducing a simple lag-time parameter poorly described the absorption phase of salbutamol. With the transit compartment model, the BICc was significantly lower, and the GOF improved compared to the simple lag-time model.

To the best of our knowledge, no popPK studies have been conducted for oral salbutamol; only models for inhaled salbutamol have been published. Salbutamol is available in various formulations and routes of administration, as we previously stated, and although the primary route for delivering anti-asthmatic drugs is inhalation, it is essential to study the influence of covariates on the PK of alternative administration routes from the perspective of individualized treatment. Incorporating covariates into the final popPK model enabled quantification of the roles of gender, body weight, BSA, and health status on relevant PK metrics.

The current research population included individuals aged between 5 and 65 years, with an average age of 39.5 years. The results indicate that age does not affect the PK parameters of orally administered salbutamol. In contrast, when administered via inhalation, age appears to have a significant effect on drug elimination, as demonstrated in our previous work [20]. Another study conducted by Bønnelykke et al. [20], which explored age dependency in systemic exposure of salbutamol administered through a spacer (inhaler), showed that serum concentration in younger children is much slower than in older children, suggesting the need for dose adjustment, otherwise, treatment may be suboptimal. Thus, the fact that oral salbutamol kinetics are not altered by age could favor the prescription of the oral regimen for children, especially for those who may face challenges in effectively using inhalers.

The proportion of females and males in this study was, respectively, 40% and 60%. Covariate analysis shows that gender impacts C*l*. In Figure 6a, a steeper decline in the terminal phase of the salbutamol PK profile can be observed in females compared to males, suggesting that the drug is being removed from the body at a higher rate. Metabolic differences are the major cause of differential PK between males and females [21,22]. The activity of some metabolic enzymes is often increased in one gender. For example, CYP2D6, a metabolizing enzyme for salbutamol, displays higher activity in females [23], which could explain the greater C*l*. However, CYP2D6 expression, as well as CYP2C19 expression, were examined as covariates, showing no influence on the PK parameters of this β_2_-agonist. Another hypothesis to explain these variations is anatomical differences (e.g., body composition) [24,25]. On the other hand, gender also significantly influences the M_tt_ for the absorption compartment. In this context, the geometric mean for females was 0.21 h, while for males it was 0.30 h. These values are consistent with the K_a_ values which is a parameter indirectly affected by the M_tt_. With a lower M_tt_, meaning the time required for the compound to move through the transit compartments and reach systemic circulation is shorter, absorption is faster.

Additionally, weight and BSA were found to affect both V1 and C*l*. Despite these two covariates being correlated, they capture complementary physiological aspects—weight as an indicator of mass and BSA as an indicator of surface area—that contribute uniquely to the IIV in salbutamol PK parameters. In populations with a wide range of body compositions (26 kg to 109 kg; 0.94 m^2^ to 2.37 m^2^), such as this virtual cohort, accounting for body weight and BSA may offer a more accurate depiction of drug disposition. Weight and BSA are metrics commonly used in drug dosing, assuming that distribution and elimination increase proportionally with these measures [26]. However, this assumption often leads to either overestimated or underestimated dosing, as it may not fully account for individual variability in metabolic and distributional capacity. BSA is widely applied in medical fields such as chemotherapy, transplantation, burn treatment, and toxicology [27]. As far as we know, no bronchodilator oral treatments are currently based on BSA or body weight.

The stratified analysis of C*l* and V1 based on weight and BSA (Table 7) reveals that, for the 26–75 kg with a BSA < 1.6 m^2^ group, C*l* was observed to be 78 L/h, and V1 was 226 L. In comparison, within the same weight range but with BSA > 1.6 m^2^, C*l* decreased to 69 L/h, and V1 was increased to 276 L. This trend suggests that individuals with a higher BSA may experience an increase in V1, due to the physiological distribution differences conferred by larger surface area and tissue composition. A higher BSA generally correlates with greater extracellular fluid volume and potentially increased organ size, both of which can augment V_d_ for a hydrophilic drug like salbutamol. On the other hand, the observed reduction in C*l* suggests that metabolic and renal C*l* processes may vary significantly across body compositions.

Our findings suggest that weight also influences C*l* and V1, with individuals > 75 kg (considered overweight and obese) showing relatively lower C*l* and higher V1 than those < 75 kg. Additionally, we observed a negative correlation between weight and elimination rate (see Supplementary Material, Figure S3), implying that an increase in weight is associated with a decrease in salbutamol elimination rate. Higher weight is typically linked to greater adipose tissue mass and volume, which results in an expanded lipophilic compartment [28]. Given the polar nature of salbutamol, it was expected that increased adipose mass might reduce the drug’s diffusion into these tissues—a finding supported in V2 rather than V1. The impact of weight-related physiological changes on PK, especially on distribution, is complex and non-linear, requiring consideration of multiple factors: lean body mass proportion, body fat proportion, and total body water content. These physiological characteristics vary substantially between individuals. Gouju et al. [28] provide valuable insights into PK in obese adults. Indeed, these indicators should have been included in the modeling to better assess the influence of weight on the compound elimination. Notwithstanding, these results underscore the relevance of dose adjustment in special populations, such as obese patients.

Regarding C*l*, although data on CYP enzyme activity in obese populations remain limited, some studies have reported increased CYP2D6 and reduced CYP2C19 activities—salbutamol’s metabolizing enzymes. Moreover, salbutamol undergoes other metabolic pathways, such as glucuronic conjugation. Brill et al. [29] evidenced increased glucuronic conjugation activity in obese patients, which would theoretically explain an increase in C*l* in this cohort. In contrast, our results show lower C*l*. Since oral salbutamol is also renal excreted, obesity-related comorbidities (e.g., diabetes, hypertension) associated with impaired renal function [28] may explain this finding. Although our popPK model indicates considerable variability in how BSA and weight impact the PK of orally administered salbutamol in the studied population, these results were not filtered by age group, which may have influenced the outcomes. Further studies are warranted to quantify the role of BSA and weight in driving salbutamol PK variability.

Furthermore, this study aimed to include an equitable proportion of different races—American, Chinese, and Asian—to investigate the influence of this covariate on the oral salbutamol profile and to address gaps found in previous clinical trials. Race was not included as a covariate in the model, as no significant statistical correlations with PK parameters were found. However, PK variations caused by race have been reported for salbutamol DPI [14], likely due to previously identified polymorphisms. Again, the choice of salbutamol formulation could consider race if it is found to be a factor contributing to differences in the drug’s profile for a given patient.

Health status, subdivided into healthy (non-obese, no comorbidities), obese, and cirrhosis A groups, was found to impact C*l* as well. Obese individuals demonstrated lower C*l* values, which is consistent with our findings from the weight analysis. On average, patients with cirrhosis A showed higher C*l* values than healthy subjects. Cirrhosis is a liver condition characterized by fibrosis (scar tissue), which replaces functioning tissue due to chronic liver disease [30]. Cirrhotic patients are categorized according to the Child–Pugh (CP) scoring system, designed to predict mortality risk: A—good hepatic function, B—moderately impaired hepatic function, and C—advanced hepatic dysfunction [31]. In this study, only patients in the early stage of cirrhosis (CP = A) were included; hence, the numerical difference between the mean C*l* in cirrhotic and healthy patients is not that large. In theory, CYP enzyme activity and hepatic blood flow are reduced in these patients, leading to increased drug exposure and heightened risk of toxicity [32,33]. Duthaler et al. [32] demonstrated that dose adjustment is crucial in this group of patients due to the increased risk of adverse drug reactions (ADRs) and hospitalizations. Given that salbutamol undergoes hepatic C*l*, it may also require careful re-evaluation in cirrhotic patients. Further studies are needed to better understand the impact of administering a bronchodilator in patients with impaired liver function.

Despite the discouragement of oral salbutamol prescriptions by prominent asthma guidelines authorities [4], such as the Global Initiative for Asthma (GINA), studying the influence of patient characteristics on different available formulations remains important from a personalized medicine perspective. According to GINA guidelines [34], “oral bronchodilator therapy is not recommended due to its slow onset of action and a higher rate of adverse effects compared to inhaled SABAs”. Nevertheless, this study suggests a potential bias in detecting adverse effects of salbutamol, as individual characteristics may indeed contribute to increased salbutamol toxicity risk. Weight, BSA, and gender are the identified covariates that significantly impact drug disposition, leading to an elevated risk of toxicity and, consequently, a higher risk of ADRs. To mitigate these risks in clinical practice, strategies such as therapeutic drug monitoring (TDM) could be employed to ensure that plasma salbutamol concentrations remain within the therapeutic window, particularly for at-risk populations and in those for whom alternative drug delivery routes are not feasible. Dose optimization and individualized treatment strategies, as advocated by the authors, should prioritize minimizing the risk of ADRs. Thus, by identifying subpopulations at higher risk of experiencing adverse effects, targeted therapy can be implemented using alternative drug delivery methods, or dose adjustments while maintaining oral therapy when other methods are not viable for prescription in specific patients.

## 5. Conclusions

This popPK study, including gender, weight, BSA, and health status as covariates, further underscores the potential of popPK modeling for personalized therapy, where drug prescriptions are informed not only by disease pathophysiology but also by patient-specific physiological, anatomical, and demographic factors. Individualized treatment brings us one step closer to improved therapeutic outcomes. Notwithstanding, the current model has inherent limitations that should be acknowledged. One key source of uncertainty arises from the reliance on in silico predictions, given the scarcity of reported PK data for oral salbutamol. Although these predictions provide a foundational framework, they may not fully capture the variability observed in diverse patient populations. Additionally, the lack of genetic polymorphism data, particularly for CYP2D6 and CYP2C19 enzymes, may have limited the model’s ability to account for IIV in drug metabolism. Another potential source of error lies in the absence of other potentially significant factors, such as comorbidities or concurrent medications, which may have introduced additional variability into the model. Therefore, since this work represents a preliminary step, complementary PD studies should be performed to assess therapeutic response and confirm these findings. The exploration of the PK/PD relationship is indeed crucial since the identified PK variability in oral salbutamol could potentially leads to differences in PD responses across individuals. In patients with altered metabolism or distribution, higher or lower drug concentrations may result in insufficient bronchodilation or increased risk of toxicity and ADRs. In addition, future studies focusing on validating this model using data from clinical trials or observational real-world cohorts should be conducted.

## Figures and Tables

**Table 1:** Input physicochemical properties of salbutamol.

Parameter	Value	Reference
Molecular weight (g/mol)	239.32	DrugBank
LogP	0.76	ADMET Predictor
pKa	9.34 (basic); 10.52 (acidic)	ADMET Predictor
Solubility (mg/mL)	14.47 @ pH 9.93	ADMET Predictor
Diff. coeff. (cm^2^ × 10^5^)	0.804	ADMET Predictor
R_bp_	1.108	ADMET Predictor
P_eff_ (cm/s 10^−4^)	1.21	ADMET Predictor
f_up_ (%)	79.65	ADMET Predictor

LogP—octanol/water partition coefficient; pKa—acid dissociation constant; Diff. coeff.—diffusion coefficient; R_bp_—blood-to-plasma concentration ratio; P_eff_—effective permeability; f_up_—fraction unbound in plasma.

**Table 2:** System-specific input parameters regarding tissue:plasma partition coefficients (K_p_).

Tissue	K_p_
Liver	9.01 ^a^
Spleen	6.70 ^a^
Muscle	3.27 ^a^
Heart	3.01 ^b^
Brain	3.01 ^b^
Adipose	0.55 ^b^
Lung	7.83 ^a^
Kidney	3.01 ^b^
Skin	3.21 ^a^
Bone	2.27 ^b^
Reproductive organs	9.75 ^a^
Rest of body	6.70 ^a^

^a^ Estimated by ADMET Predictor^®^, ^b^ extracted from Boger et al. [15].

**Table 3:** Input pharmacokinetic properties of salbutamol.

Parameter	Value	Note
F_a_ (%)	85.20	Derived from ADMET Predictor
F (%)	52.41	Derived from ADMET Predictor
V_d_ (L)	167.02	Optimized value. Reference value: 195.75 for a 75 kg man (2.61 L/kg)
C_max_ (ng/mL)	7.250	Derived from ADMET Predictor
T_max_ (h)	2.35	Derived from ADMET Predictor
AUC (ng·h/mL)	31.75	Derived from ADMET Predictor
C*l* (L/h)	Hepatic C*l* = 38.06 Renal C*l* = 16.32	Optimized value. Reference value for C*l*: 46.38
t_1/2_ (h)	2.78	Optimized value. Reference value: 3.36

F_a_, fraction absorbed; F, bioavailability; V_d_, volume of distribution, C_max_, maximum concentration, T_max_, time to reach maximum concentration, C*l*, clearance, t_1/2_, half-life.

**Table 4:** Observed (reference) and simulated PK parameters for a single oral dose of salbutamol.

	F_a_ (%)	F (%)	C_max_ (μg/mL)	T_max_ (h)	AUC_inf_ (ng·h/mL)
Observed	85.2	52.4	0.00725	2.35	0.0318
Predicted	87.7	47.4	0.00919	1.28	0.0553
Fold-error	1.03	1.11	1.27	1.84	1.74

F_a_, fraction absorbed; F, bioavailability; C_max_, maximum concentration; T_max_, time to reach C_max_; AUC_inf_, area under the concentration–time curve extrapolated to infinity.

**Table 5:** Demographic and clinical characteristics of patients (mean or median ± standard deviation, SD, or interquartile range, IR).

Characteristics	Total (*n* = 40)
Age (years)	39.5 ± 18.4
Gender (*n*, %)	
Female	16, 40
Male	24, 60
BSA (m^2^)	1.64 ± 0.330
Weight (kg)	60.2 ± 19.8
CYP2C19 expression (%)	1.30 ± 1.50
CYP2D6 expression (%)	1.90 ± 1.60
Race (*n*, %)	
American	20, 50
Asian	10, 25
Chinese	10, 25
Health status (*n*, %)	
Healthy	26, 65
Obese	4, 10
Cirrhosis A	10, 25

**Table 6:** Parameter estimates for the final model.

Parameters	Estimate	RSE (%)
Fixed effects		
M_tt_ (h)	9.50	7.14
K_tr_ (h^−1^)	0.15	9.21
k_a_ (h^−1^)	2.91	6.80
C*l* (L/h)	140	223
V1 (L)	77.5	97.2
Q (L/h)	48.9	24.9
V2 (L)	130	21.4
Random effects		
IIV (M_tt_)	0.43	13.0
IIV (K_tr_)	0.32	13.8
IIV (k_a_)	0.41	15.4
IIV(C*l*)	0.38	13.0
IIV (V1)	0.36	12.3
IIV (Q)	0.92	14.9
IIV(V2)	0.87	27.4
Error model parameters		
*a*	$0.19\times 10^{-4}$	20.2
*b*	$0.15\times 10^{-2}$	37.2

RSE, relative standard error; M_tt_, mean transit time; K_tr_, transit rate; k_a_, absorption constant rate; C*l*, clearance; V1, volume of distribution of the central compartment; V2, volume of distribution of the second (peripheral) compartment; Q, intercompartmental clearance; IIV, interindividual variability; *a* and *b*, constants of the error model.

**Table 7:** PK parameters of salbutamol across different patient subgroups defined by gender, BSA, and weight. For the M_tt_, values are shown for male and female groups. For the V1 and C*l*, values are stratified according to BSA and weight categories. The geometric mean and SD are provided for each subgroup.

Parameter	Covariate		Geometric Mean	SD
M_tt_	Gender	Female	0.21	3.89
		Male	0.30	1.37
	BSA	Weight		
V1	0.94–1.60	26.4–75.0	226	1.56
	0.94–1.60	26.4–75.0	276	1.54
	1.60–2.37	75.0–109	287	1.84
Cl	0.94–1.60	26.4–75.0	77.9	1.51
	0.94–1.60	26.4–75.0	69.9	2.28
	1.60–2.37	75.0–109	68.2	1.45

## Data Availability

The original contributions presented in this study are included in the article/supplementary material. Further inquiries can be directed to the corresponding authors.

## Supplementary Materials

The following supporting information can be downloaded at: https://www.mdpi.com/article/10.3390/pharmaceutics17010039/s1, Table S1. Key physicochemical and pharmacokinetic properties used as input for the PBPK model; Table S2. Detailed demographic and clinical characteristics of the virtual population generated from PBPK modeling; Table S3. PK parameters obtained in the virtual population following oral treatment with 4 mg q6h of salbutamol. Values are presented as geometric mean and coefficient of variation (CV%); Table S4. Basic popPK models of salbutamol; Table S5. Statistical analysis of correlations between covariates and PK parameters; Figure S1. Correlation analysis between age and body surface area (BSA) with (a) area under the curve (AUC_0-inf_), (b) maximum concentration (C_max_), (c) apparent clearance (C*l*/F), (d) elimination rate (k_el_), (e) time to reach C_max_ (T_max_), and (f) apparent volume of distribution (V_d_/F). A strong correlation is observed between BSA and k_el_ (correlation: –0.69); Figure S2. Correlation analysis between CYP2C19 and CYP2D6 expression with (a) area under the curve (AUC_0-inf_), (b) maximum concentration (C_max_), (c) apparent clearance (C*l*/F), (d) elimination rate (k_el_), (e) time to reach C_max_ (T_max_), and (f) apparent volume of distribution (V_d_/F). A strong correlation is observed between BSA and k_el_ (correlation: –0.69); Figure S3. Correlation analysis between weight and gender with (a) area under the curve (AUC_0-inf_), (b) maximum concentration (C_max_), (c) apparent clearance (C*l*/F), (d) elimination rate (k_el_), (e) time to reach C_max_ (T_max_), and (f) apparent volume of distribution (V_d_/F). A strong correlation is observed between weight and k_el_ (correlation: –0.64), and gender and k_el_; Figure S4. Correlation analysis between health status and race with (a) area under the curve (AUC_0-inf_), (b) maximum concentration (C_max_), (c) apparent clearance (C*l*/F), (d) elimination rate (k_el_), (e) time to reach C_max_ (T_max_), and (f) apparent volume of distribution (V_d_/F). Health status is a covariate with a potential effect on C*l*. Race has a significant impact on k_el_.
