# Supplementary material for: Improving Individualized Salbutamol Treatment: A Population Pharmacokinetic Model for Oral Salbutamol in Virtual Patients
Source: Pharmaceutics. 2024 Dec 30;17(1):39. doi: 10.3390/pharmaceutics17010039 (PMC11768577; doi:10.3390/pharmaceutics17010039)
Supplement: Supplementary file 1 [file pharmaceutics-17-00039-s001.zip › pharmaceutics-3377815-supplementary.pdf]

Supplementary Materials: Optimizing Salbutamol Treatment: The Critical Role of Patient Covariates in Drug Formulation

Lara Marques [1,2,3] and Nuno Vale [1,2,3]

1 PerMed Research Group, Center for Health Technology and Services Research (CINTESIS), Rua Doutor Plácido da Costa, 4200-450 Porto, Portugal; lara.marques2010@hotmail.com
2 CINTESIS@RISE, Faculty of Medicine, University of Porto, Al. Prof. Hernâni Monteiro, 4200-319 Porto, Portugal
3 Department of Community Medicine, Health Information and Decision (MEDCIDS), Faculty of Medicine, University of Porto, Rua Dr. Plácido da Costa, 4200-450 Porto, Portugal
* Correspondence: nunovale@med.up.pt; Tel.: +351-220426537

1. PBPK Model Development

Table S1. Key physicochemical and pharmacokinetic properties used as input for the PBPK model.

Parameter	Value	Notes
Mean precipitation time (s)	900	GastroPlus default
Drug particle density (g/mL)	1.2	GastroPlus default
Particle radius (µm)	25	Not relevant as the drug is delivered in solution, not as particles; GastroPlus default
Gut physiology	Human-physiological-fasted	
Absorption model ASF (cm^{-1})	Duodenum = 2.710<br>Jejunum 1 = 2.660<br>Jejunum 2 = 2.661<br>Ileum 1 = 2.624<br>Ileum 2 = 2.612<br>Ileum 3 = 2.582<br>Caecum = 0.019<br>Ascending colon = 0.046	Human-physiological-fasted OptlogD model SA/V 6.1, used to scale passive effective permeability across different intestinal regions, adjusting for variations in the surface:volume ratio and pH along the GI tract
Intestinal FPE	35%	Fitted to PO PK data
Kp prediction method	Lukacova (Rodgers and Rowland method)	Perfusion-limited model

ND, not defined; ASF, absorption scale factor; GI, gastrointestinal; FPE, first-pass extraction; PO, oral; PK, pharmacokinetic; Kp, tissue:plasma partition coefficient.

Table S2. Detailed demographic and clinical characteristics of the virtual population generated from PBPK modeling.

Group	Race	Gender	Age (years)	Health Status	Weight (kg)	Body Surface Area (cm^2)	CYP2C19 Expression	CYP2D6 Expression
	American	Male	29	Healthy	91.59	2.13	0.057	0.020
	American	Male	33	Healthy	52.90	1.50	0.018	0.016
1	American	Male	62	Obese	109.5	2.37	0.014	0.008
	American	Male	41	Healthy	91.10	2.09	0.013	0.038
	American	Female	9	Obese	38.04	1.13	0.052	0.012

	American	Female	63	Healthy	58.09	1.58	0.016	0.034
	American	Female	18	Healthy	69.41	1.76	0.026	0.023
	American	Female	9	Obese	33.60	1.05	0.013	0.007
	American	Female	20	Obese	94.37	2.05	0.050	0.023
	American	Male	31	Healthy	74.38	1.92	0.046	0.023
2	Chinese	Male	67	Healthy	50.50	1.50	0.013	0.030
	Chinese	Female	43	Healthy	56.86	1.53	0.031	0.010
	Chinese	Female	48	Healthy	63.47	1.65	0.057	0.023
	Chinese	Female	44	Healthy	59.55	1.59	0.022	0.017
	Chinese	Female	67	Healthy	63.08	1.67	0.022	0.020
	Chinese	Male	8	Healthy	29.10	1.05	0.013	0.032
	Chinese	Male	43	Healthy	57.22	1.60	0.028	0.051
	Chinese	Female	6	Healthy	26.40	0.95	0.013	0.015
	Chinese	Female	51	Healthy	54.19	1.56	0.023	0.049
	Chinese	Male	44	Healthy	64.49	1.69	0.005	0.019
3	Asian	Male	65	Healthy	68.85	1.80	0.003	0.019
	Asian	Male	31	Healthy	55.55	1.60	0.003	0.097
	Asian	Male	34	Healthy	51.85	1.63	0.015	0.017
	Asian	Female	41	Healthy	54.81	1.49	0.002	0.036
	Asian	Male	53	Healthy	50.83	1.41	0.021	0.024
	Asian	Male	38	Healthy	56.48	1.58	0.012	0.018
	Asian	Female	21	Healthy	52.01	1.45	0.008	0.034
	Asian	Male	47	Healthy	47.93	1.43	0.004	0.023
	Asian	Female	20	Healthy	48.38	1.52	0.005	0.024
	Asian	Male	66	Healthy	60.77	1.66	0.013	0.032
4	American	Female	35	Cirrhosis A	92.09	2.15	0.005	0.010
	American	Male	22	Cirrhosis A	97.58	2.19	0.015	0.005
	American	Male	43	Cirrhosis A	84.07	2.11	0.004	0.004
	American	Male	22	Cirrhosis A	73.41	1.83	0.012	0.009
	American	Male	69	Cirrhosis A	95.42	2.15	0.023	0.013
	American	Male	47	Cirrhosis A	83.06	2.03	0.011	0.013
	American	Male	31	Cirrhosis A	72.93	1.82	0.009	0.016
	American	Male	20	Cirrhosis A	90.37	2.09	0.003	0.010
	American	Female	66	Cirrhosis A	58.05	1.57	0.015	0.024
	American	Male	26	Cirrhosis A	73.70	1.78	0.011	0.008

2. Virtual Dataset Generation

Table S3. PK parameters obtained in the virtual population following oral treatment with 4 mg q6h of salbutamol. Values are presented as geometric mean and coefficient of variation (CV%).

Group	F_a (%)	F (%)	C_{max} (µg/mL)	T_{max} (h)	AUC_{inf} (ng.h/mL)
1	78.3 ± 20.8	40.9 ± 18.5	0.0134 ± 45.3	19.2 ± 0.929	0.212 ± 40.3
2	83.3 ± 13.9	41.0 ± 18.0	0.0151 ± 46.8	19.0 ± 1.19	0.222 ± 48.7
3	80.5 ± 22.3	39.6 ± 18.6	0.0139 ± 24.8	18.8 ± 1.87	0.177 ± 35.2
4	83.6 ± 12.6	46.0 ± 18.8	0.0125 ± 20.3	19.0 ± 0.932	0.199 ± 24.9

F_a, fraction absorbed; F, bioavailability; C_{max}, maximum concentration; T_{max}, time to reach C_{max}; AUC_{inf}, area under the concentration-time curve extrapolated to infinity.

3. Structural popPK Model Building

Table S4. Basic popPK models of salbutamol.

Project Name	Model Description	Error Model	– 2LL	BICc
PK_S_01	One-compartment model, with no delay, first-order absorption, and linear elimination	Combined 1	–8516.16	–8471.68
PK_S_02	Two-compartment model, with no delay, first-order absorption, and linear elimination	Combined 1	–8668.90	–8603.68
PK_S_03	Three-compartment model, with no delay, first-order absorption, and linear elimination	Combined 1	–8560.14	–8474.18
PK_S_04	One-compartment model, with lag time, first-order absorption, and Michaelis-Menten elimination	Combined 1	–9532.8	–9477.95
PK_S_05	Two-compartment model, with lag time, first-order absorption, and linear elimination	Combined 1	–10009.34	–9933.75
PK_S_06	Three-compartment model, with lag time, first-order absorption, and linear elimination	Combined 1	–10188.40	–10092.07
PK_S_07	Two-compartment model, with transit compartment, first-order absorption, and linear elimination	Combined 1	–10045.39	–9980.17
PK_S_08	Three-compartment model, with transit compartment, first-order absorption, and linear elimination	Combined 1	–12022.77	–11936.81

–2LL, –2 × log likelihood, BICc, Corrected Bayesian Information Criterion

4. Covariate Analysis: Manual Approach

Table S5. Statistical analysis of correlations between covariates and PK parameters.

	M_{tt}	K_{tr}	k_a	*Cl*	**V1**	**Q**	**V2**
Age	0.038 (0.815)	–0.072 (0.659)	–0.027 (0.866)	–0.023 (0.886)	0.245 (0.128)	–0.106 (0.514)	–0.215 (0.182)
BSA	0.194 (0.231)	–0.261 (0.104)	–0.020 (0.093)	–0.075 (0.646)	0.510 (< 0.001)	0.071 (0.663)	–0.090 (0.583)
Weight	0.183 (0.259)	–0.262 (0.103)	–0.026 (0.873)	–0.058 (0.724)	0.516 (< 0.001)	0.097 (0.550)	–0.098 (0.549)
CYP2C19 Expression	–0.131 (0.420)	0.193 (0.234)	–0.063 (0.698)	0.130 (0.425)	–0.199 (0.219)	0.057 (0.727)	0.032 (0.845)
CYP2D6 Expression	–0.170 (0.293)	0.169 (0.299)	0.150 (0.354)	–0.063 (0.700)	–0.202 (0.211)	0.134 (0.411)	–0.149 (0.359)
Gender	4.378 (0.043)	1.989 (0.167)	0.115 (0.737)	4.828 (0.034)	0.168 (0.684)	0.982 (0.328)	0.769 (0.386)
Health Status	0.530 (0.593)	0.534 (0.591)	0.770 (0.470)	0.492 (0.615)	1.422 (0.254)	0.419 (0.661)	0.067 (0.935)
Race	2.850 (0.071)	2.404 (0.104)	2.501 (0.096)	0.248 (0.782)	1.422 (0.254)	0.477 (0.624)	0.895 (0.417)

CYP, cytochrome P450; M_{tt}, mean transit time; K_{tr}, transit rate; k_a, absorption constant rate; *Cl*, clearance; V1, volume of distribution of the central compartment; Q, intercompartmental clearance; V2, volume of distribution of the peripheral compartment.

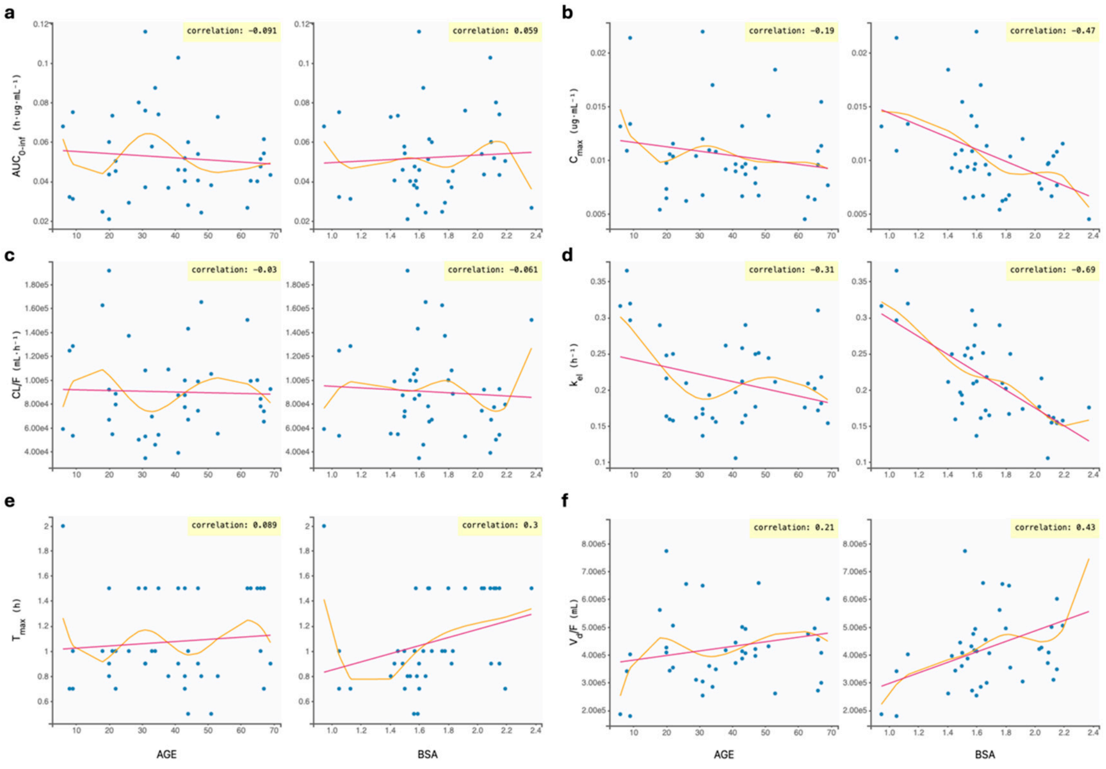

Figure S1. Correlation analysis between age and body surface area (BSA) with (a) area under the curve ($AUC_{0\text{-inf}}$), (b) maximum concentration (C_{max}), (c) apparent clearance (*Cl*/F), (d) elimination rate (k_{el}), (e) time to reach C_{max} (T_{max}), and (f) apparent volume of distribution (V_d/F). A strong correlation is observed between BSA and k_{el} (correlation: –0.69).

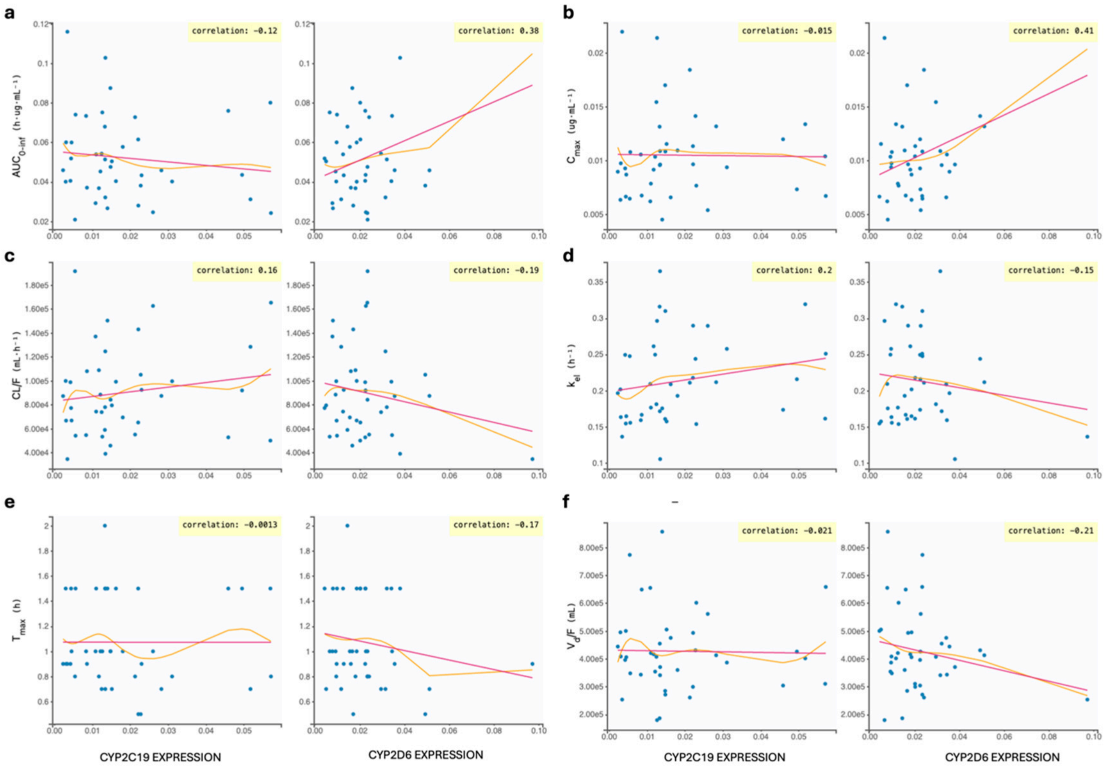

Figure S2. Correlation analysis between CYP2C19 and CYP2D6 expression with (a) area under the curve ($AUC_{0\text{-}inf}$), (b) maximum concentration (C_{max}), (c) apparent clearance (C*l*/F), (d) elimination rate (k_{el}), (e) time to reach C_{max} (T_{max}), and (f) apparent volume of distribution (V_d/F). A strong correlation is observed between BSA and k_{el} (correlation: –0.69).

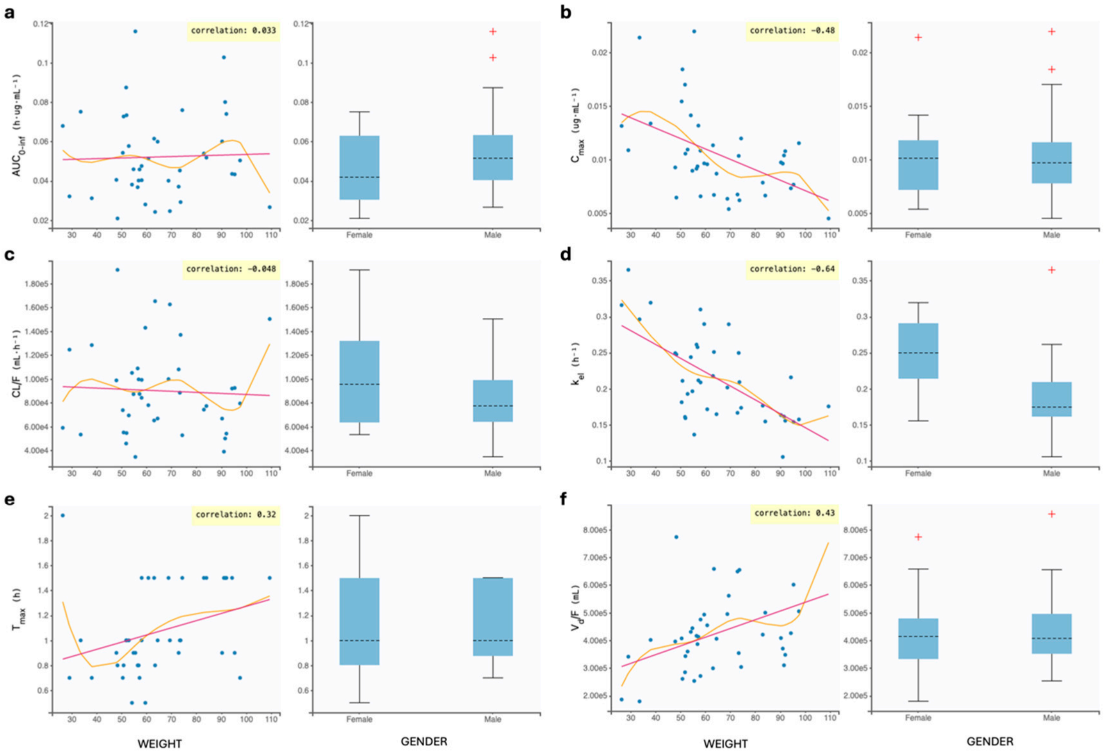

Figure S3. Correlation analysis between weight and gender with (a) area under the curve ($AUC_{0\text{-}inf}$), (b) maximum concentration (C_{max}), (c) apparent clearance (C*l*/F), (d) elimination rate (k_{el}), (e) time to reach C_{max}

(T_{max}), and (f) apparent volume of distribution (V_d/F). A strong correlation is observed between weight and k_{el} (correlation: –0.64), and gender and k_{el}.

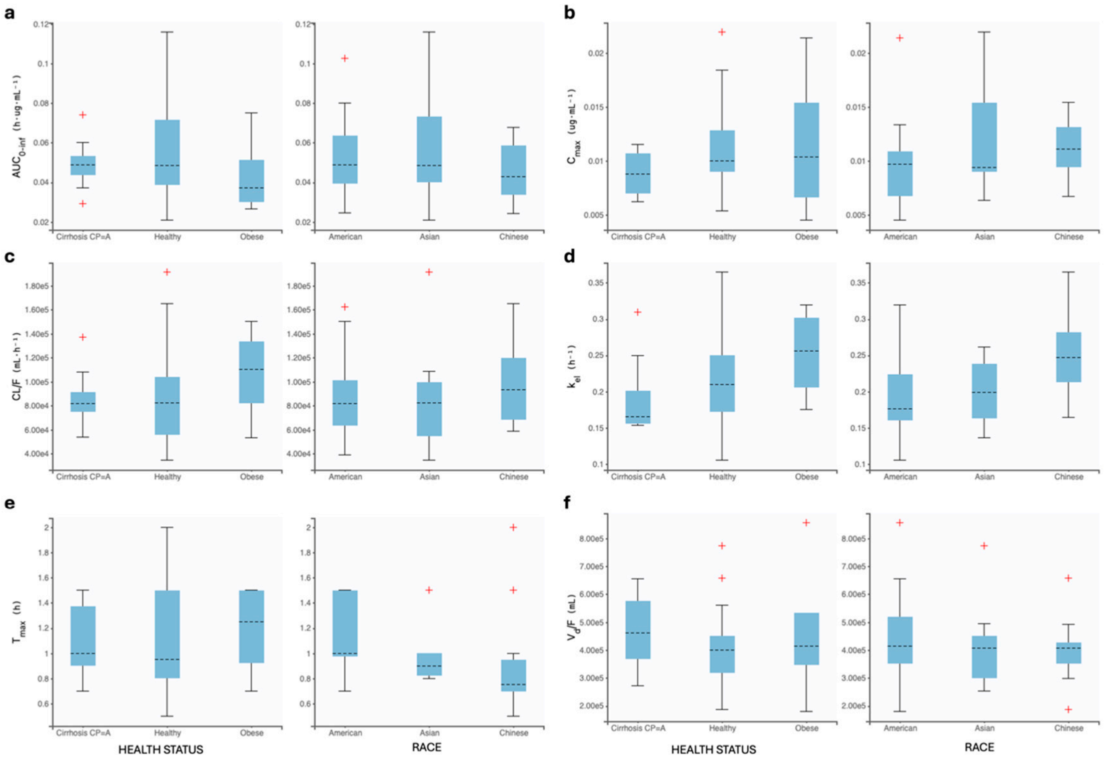

Figure S4. Correlation analysis between health status and race with (a) area under the curve ($AUC_{0\text{-}inf}$), (b) maximum concentration (C_{max}), (c) apparent clearance (Cl/F), (d) elimination rate (k_{el}), (e) time to reach C_{max} (T_{max}), and (f) apparent volume of distribution (V_d/F). Health status is a covariate with a potential effect on C*l*. Race has a significant impact on k_{el}.
